# Functional consultation and exercises improve grip strength in osteoarthritis of the hand – a randomised controlled trial

**DOI:** 10.1186/s13075-018-1747-0

**Published:** 2018-11-09

**Authors:** Michaela A. Stoffer-Marx, Meike Klinger, Simone Luschin, Silvia Meriaux-Kratochvila, Monika Zettel-Tomenendal, Valerie Nell-Duxneuner, Jochen Zwerina, Ingvild Kjeken, Marion Hackl, Sylvia Öhlinger, Anthony Woolf, Kurt Redlich, Josef S. Smolen, Tanja A. Stamm

**Affiliations:** 1 Section for Outcomes Research, Center for Medical Statistics, Informatics, and Intelligent Systems, Medical University of Vienna, Spitalgasse 23, 1090 Vienna, Austria; 2 Division of Rheumatology, Department of Medicine 3, Medical University of Vienna, Vienna, Austria; 3 Department of Health Sciences, University of Applied Sciences FH Campus Wien, Favoritenstraße 226, 1100 Vienna, Austria; 4 Physio Austria, Lange Gasse 30/1, Vienna, Austria; 5 Klinikum Peterhof of NOEGKK with Ludwig Boltzmann Department of Epidemiology of Rheumatic Diseases, Sauerhofstraße 9-15, Baden bei Wien, Austria; 6 Ludwig Boltzmann Institute of Osteology, 1st Medical Department at Hanusch Hospital, Hanusch Hospital of the WGKK and AUVA Trauma Center, Heinrich Collin Str. 30, 1140 Vienna, Austria; 7 National advisory unit on rehabilitation in rheumatology, Department of rheumatology, Diakonhjemmet Hospital, Oslo, Norway; 8 Program of Occupational Therapy, Prosthetics and Orthotics, Oslo Metropolitan University, Oslo, Norway; 9 Ergotherapie Austria, Bundesverband der Ergotherapeutinnen und Ergotherapeuten Österreichs, Holzmeistergasse 7-9/2/1, Vienna, Austria; 10 University of Applied Sciences for Health Professions Upper Austria, Semmelweisstraße 34, Linz, Austria; 11 Bone and Joint Research Group, Royal Cornwall Hospital, Truro, UK

**Keywords:** Osteoarthritis, Hand, Occupational therapy, Physiotherapy, Pain management, Quality of healthcare

## Abstract

**Background:**

Evidence for non-pharmacological interventions in hand osteoarthritis is promising but still scarce. Combined interventions are most likely to best cover the clinical needs of patients with hand osteoarthritis (OA). The aim of this study was to evaluate the effect of a combined, interdisciplinary intervention feasible in both primary and specialist care compared to routine care plus placebo in patients with hand OA.

**Methods:**

This was a randomised, controlled 2-month trial with a blinded assessor. In the combined-intervention group, rheumatology-trained health professionals from different disciplines delivered a one-session individual intervention with detailed information on functioning, activities of daily living, physical activity, nutrition, assistive devices, instructions on pain management and exercises. Telephone follow up was performed after 4 weeks. The primary outcome was grip strength after 8 weeks. Secondary outcomes were self-reported pain, satisfaction with treatment, health status, two of the Jebsen-Taylor Hand Function subtests and the total score of the Australian/Canadian Hand Osteoarthritis Index (AUSCAN). Statistical significance was calculated by Student’s *t* test or the Mann-Whitney U test depending on data distribution. Binominal logistic regression models were fitted, with the primary outcome being the dependent and the group allocation being the independent variable.

**Results:**

There were 151 participating patients (74 in the combined-intervention and 77 in the routine-care-plus-placebo group) with 2-month follow-up attendance of 84% (*n* = 128). Grip strength significantly increased in the combined-intervention group and decreased in the routine-care group (dominant hand, mean 0.03 bar (SD 0.11) versus − 0.03 (SD 0.13), *p* value = 0.001, baseline corrected values) after 8 weeks.

**Conclusion:**

The combined one-session individual intervention significantly improved grip strength and self-reported satisfaction with treatment in patients with hand OA. It can be delivered by different rheumatology-trained health professionals and is thus also feasible in primary care.

**Trial registration:**

ISRCTN registry, ISRCTN62513257. Registered on 17 May 2012.

**Electronic supplementary material:**

The online version of this article (10.1186/s13075-018-1747-0) contains supplementary material, which is available to authorized users.

## Key message

A combined intervention feasible in primary and specialist care improves grip strength.

## Background

The prevalence of rheumatic diseases rises with age and with increasing longevity of the population [1]. Osteoarthritis (OA) is one of the most common rheumatic diseases and is associated with damage of the articular cartilage and changes in subchondral bone [2]. OA affects 60–70% of the population above the age of 65 years [3–5]. Today, almost 80% of the population can expect to live through most of their seventh decade of life, thus the impact of OA is likely to increase even further in the future [3]. Hand OA leads to a reduction of grip strength [6], difficulties when performing tasks of everyday life [7], loss of productive work time [8] and a decreased ability to perform manual activities [9]. Given the importance of being able to use the hands in daily life, it is apparent that hand OA affects not only body functions and structures but also several activities of daily living and societal participation [10, 11]. Thus, hand OA is a burden not only for the individual but also for society [12].

Management recommendations advise applying pharmacological and non-pharmacological methods for hand OA [13]. Drug treatment recommended for hand OA includes analgesics, non-steroidal anti-inflammatory drugs (NSAIDs) and topical active components [14]. Patients with OA, including hand OA, are frequently managed in primary care and are commonly referred to non-physician health professionals such as occupational therapists and physiotherapists to improve their health and functional performance [13, 15–17].

Based on existing guidelines, patient perspectives and expert opinion, the eumusc.net-working group developed user-focused European standards of care for OA [18]. From a recent focus group study also undertaken within the eumusc.net project, it is known that patients wish to have the possibility to contact a health professional experienced in the care of OA directly over the phone for a follow-up consultation [19].

Recent trials on multidisciplinary interventions in hand, knee, hip [20, 21] and generalised OA [22] showed significance in the subjective self-reported satisfaction of the patients but failed to show effects on clinical outcomes [23, 24]. Furthermore, multidisciplinary approaches were tested only in group settings. Given that the number of OA patients will increase in future, time efficient and individualised treatment options that can be delivered by different health professionals are desirable in OA. As hand OA patients have individual and subjective treatment requirements, combined interventions that take different treatment options into account may be superior to interventions that focus on just one particular component, e.g. exercises only or orthoses only. However, there is a lack of evidence on these approaches, especially those feasible in primary care.

Thus, high-quality studies on the effect of a brief interdisciplinary, individualized, intervention that is community-applicable in both primary and specialist care are warranted.

The aim of the study was to compare the effect of a combined intervention feasible in both, primary and specialist care, compared with routine care plus placebo in patients with hand OA.

## Methods

### Study design

The present study was an assessor-blinded randomised controlled trial (RCT) (Table 1) to evaluate functional outcome and personal satisfaction in patients undergoing a combined interdisciplinary intervention, compared with routine clinical care plus a massage ball as placebo intervention. Patients were allocated at a rate of 1:1 to the two groups. Assessments were performed at baseline and the follow-up visit in outpatient clinics 2 months later. Principles of good clinical practice, the Declaration of Helsinki, Consolidated Standards of Reporting Trials (CONSORT) statement 2010 [25] and CONSORT guidelines for non-pharmacological interventions [26] have been taken into consideration in the planning, realisation and reporting of this trial. According to the Declaration of Helsinki the ethical committee of the Medical University of Vienna approved the study (number 1083/2012). Participants gave their written informed consent. The trial was registered at www.controlled-trials.com with the trial registration number ISRCTN62513257.

**Table 1:** Content of the combined-intervention delivered to participants according to the patient-centred standards of care for osteoarthritis [18]

Flow chart combined intervention compared to RC group	All mentioned health professionals developed the intervention together. The combined intervention was delivered by an occupational therapist, physiotherapist, dietician and/or nurse according to their availability and the needs of the patient
Pain and difficulties with ADL assessment	Patients have been asked in detail about the pain they experience and asked to report three activities of daily living that are difficult to perform for them
Information and functioning consultation	The diagnosis made by the rheumatologist was explained to the patient in an easy and lay understandable format.
	Information on an active and healthy lifestyle, physical activity, nutrition supplements, nutrition and if necessary (BMI > 27) information about the benefit of an optimal bodyweight was given
	Possibilities of surgery were discussed briefly
	Strategies for self-efficacy, ergonomic principles and pacing strategies were explained to maximise physical functioning
Pain management	Information about medication, the resulting benefit and risks according to available guidelines was given. The value of thermotherapy was explained, especially the benefit of heat during stable periods and cold during inflamed periods. A hot and cold pack to apply hot and cold packing at home was provided free of charge for the patients
Assistive devices	According to the difficulties in daily living reported by the patient, aids and devices were discussed and shown to the patient. Opening screw-caps is a commonly reported problem. If a patient mentioned having problems with this task a non-slip mat (Dycem) was provided and patients were trained in opening glasses/bottles with this device
	All patients were assessed for the need of a CMC 1 joint orthosis (Additional file 1: Material 1 pictures of the CMC 1 orthosis). If required it was custom-made during the consultation
Hand exercises	Patients in the combined-intervention group received instructions for a home-exercise programme to enhance range of motion and grip strength. Detailed information about the hand exercise programme is given in Fig. 1. The therapy putty necessary for the exercise programme was directly provided from the interdisciplinary team free of charge for the patient
	Participants were instructed to exercise daily for 8 weeks with 10 repetitions during weeks 1–2, 12 repetitions in weeks 3–4 and 15 repetitions in weeks 5–8. Beside access to a web-based interactive online video showing the exercises for the hands, the programme was given in paper format to the participants. The programme is accessible following the link: https://elearning.fh-campuswien.ac.at/WBT/Fingerpolyarthrose/2014.html
Follow up	An appointment was scheduled for 8 weeks after the baseline intervention. Patients received a telephone number/email address to contact the therapist if they need further instructions after the consultation. After 1 month, patients received a follow-up telephone call from the therapist, who asked questions according to a standardised protocol, answered emerging questions and encouraged the patient to follow the advice given and the exercise regime
	Patients were advised to bring the used therapy putty to the follow-up session in order to examine the exercise adherence of the individuals in the intervention group

Patients received a booklet with general information, exercises, contact details from the health professionals and the link for the exercise video

*ADL* activities of daily living, *BMI* body mass index, *CMC* carpometacarpal, *OT* occupational therapist, *PT* physiotherapist, *RC* routine care

### Participants

Participants diagnosed with hand OA according to the American College of Rheumatology criteria [27] were recruited from the rheumatology outpatient clinic of the Medical University in Vienna, Austria. In order to not exclude patients with hand OA at an early stage, patients with bony swelling of at least one interphalangeal joint of the second to the fifth finger and/or pain or bony swelling of at least one carpometacarpal 1 joint (CMC 1) have also been considered eligible to participate in the study. In addition, eligible patients had to score having hand pain of at least 3 points on an 11-point Likert scale at two time points, at baseline and at the intervention session.

Patients diagnosed with a rheumatic disease other than hand OA, including rheumatoid arthritis, or patients with elevated C-reactive protein levels (> 0.5 mg/dl) were excluded. Patients who had received steroid injections within the last 4 weeks, who had undergone hand surgery within the last year or who planned to receive steroid injections or surgery during the study period were also excluded.

### The combined intervention

The intervention was delivered by two rheumatology-trained health professionals who were present at the same time (either an occupational therapist, a physiotherapist, a nurse and/or a dietician), each of them having clinical expertise either in a primary or specialised care setting or both. They had to have at least 2 years of clinical experience in treating patients with rheumatic diseases. The assignment of the two health professionals was variable and depended on their availability at a specified patient visit.

**Fig. 1:**
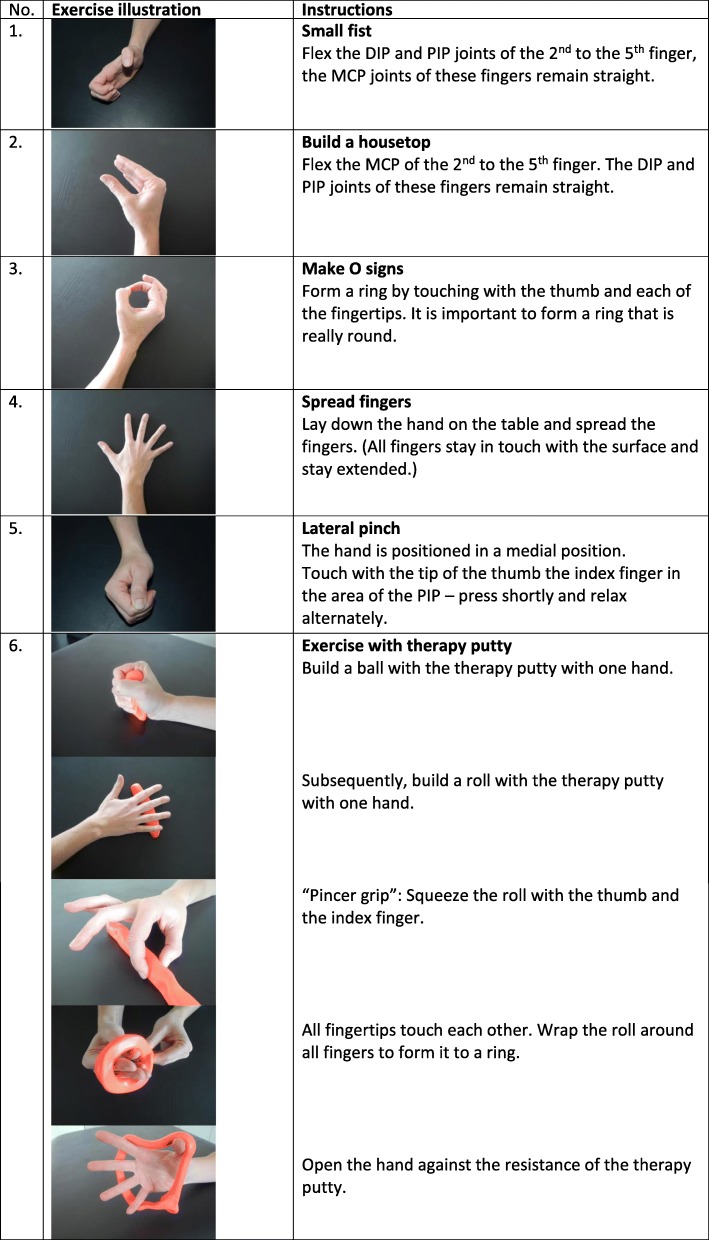
The hand exercise programme. DIP, distal interphalangeal joint; MCP, metacarpophalangeal joint; PIP, proximal interphalangeal joint

The combined intervention, which is shown in detail in Table 1, Fig. 1 and Additional file 1: Material 1, included strengthening and mobility exercises and information about physical activity; a consultation on functioning in daily life; the provision of and information on assistive devices and orthoses; information on nutrition and weight reduction, if necessary (defined by body mass index > 27), information on medication and nutrition supplements; strategies for self-efficacy, self-application of physical methods and when-to consider surgery (Table 1).

Patients in the combined intervention group were informed in detail about medications for pain management but were encouraged to use as little analgesic medication as possible. Patients were advised to try topical substances before using oral ones if they required pain medication.

After the face-to-face intervention, which was done on an individual basis, patients could contact the health professionals via email or telephone. Apart from this possibility, a structured telephone follow-up intervention in the form of a consultation was scheduled one month after the initial session. A standardised telephone protocol was used for the follow ups; additionally, patients had the opportunity to ask questions and discuss matters relevant to them during the telephone call.

### Routine care plus placebo

Patients in routine care received a massage ball as placebo intervention. Patients were instructed to roll the ball gently at the palmar and dorsal sides of their hands. In the routine care (RC) group, the decision about possible treatments or interventions was at the discretion of the rheumatologist, primary care physician or health professional seeing the patient. If considered necessary, patients were also allowed to be referred to occupational therapy, physiotherapy and a dietician for further instructions; however, these interventions were not structured according to the pre-specified protocol mentioned above. All participants in the study were allowed to use supportive medication during the trial. All interventions and visits related to the patients’ hand OA were documented during the study period.

### Assessments, endpoints and sample size calculation

Assessments were carried out at baseline and after 2 months. Participants’ sociodemographic data were obtained at baseline. Hand function is a complex concept including several motor, sensory and cognitive abilities of a person. While hand strength represents only one aspect of hand function [28], it is often used as an indicator of overall fitness and the level of physical activity. Hand strength, consisting of grip and pinch strength, is a reliable core outcome measurement for hand OA [29, 30]. Furthermore, the sensitivity to change for grip strength was found to be good [31]. We selected grip strength of the dominant hand as the primary outcome measure of our study in order to choose only one indicator. Grip strength was measured using a Martin Vigorimeter with a 43-mm rubber bulb [32, 33] (Vigorimeter, Martin Tuttlingen Germany). Three measurements were taken for both hands, and mean values were calculated for each hand.

Secondary Outcomes Were Hand Function Measured By Two Subtests Of The Jebsen-Taylor Hand Function Test (JTHFT) [34] (subtest 3 “picking up small common objects” and subtest 7 “picking up large heavy cans”), a self-report questionnaire of function - the Australian/Canadian Hand Osteoarthritis Index (AUSCAN) [35, 36] and self-reported assessment of pain and satisfaction of patients with their health care and health status (on an 11-point Likert scale). Based on results from an earlier study [15], we calculated that group sizes of 64 were needed to achieve 80% power with an alpha level of 0.05 to detect a potentially significant difference between the null hypothesis that grip strength in both groups would not significantly change and the alternative hypothesis that grip strength would change with a medium effect size (Cohen’s *d* of 0.5). We further estimated that 10% of the participants (*n* = 6) would drop out and therefore aimed at recruiting 70 participants for each group.

### Randomisation

Patients were randomised to the combined-intervention group or the RC group with an allocation ratio of 1:1 based on stratification of baseline scores for grip strength. A person from the administrative staff who neither saw patients in the clinic nor was otherwise involved in the study, performed the randomisation. To ensure comparability of the two groups, randomisation was performed in blocks of 10 patients with a similar baseline grip-strength value. The allocation of the patients was determined by a randomisation number.

### Blinding

Assessors were blinded to all details of the study. Health professionals involved in the intervention did not collect clinical data from participants in this study. According to ethical and legal regulations, patients had to be informed in detail about the study, e.g. that a patient would be randomly allocated to one of two groups. However, we did not discuss with patients our assumption that the combined intervention might be preferable when compared to the placebo intervention. The health professionals who delivered the intervention saw only those patients who had been allocated to the intervention session. Nevertheless, it is a limitation of the study that, due to the nature of the method used, patients and health professionals delivering the intervention were not fully blinded.

### Statistical methods

Descriptive statistics were used to describe the sample. Furthermore, data were assessed for normal/non-normal distribution using the Kolmogorov-Smirnov test. Differences in assessments between the groups at baseline and at month 2 were calculated by Students’ *t* test for data with normal distribution and by the Mann-Whitney U test for variables with non-normal distribution. Analyses were performed according to an intention-to-treat approach. Missing data were imputed using the method of last observation carried forward. Due to multiple testing and therefore a larger risk of type 1 error, the significance level was adjusted according to Bonferroni (*p* value of 0.05/10 outcomes) leading to a new *p* value of 0.005. We adjusted for baseline values in the analysis in that we used differences between baseline and follow-up values of grip strength for each patient.

Furthermore, we fitted logistic regression models to explore the accuracy of our results: we explored the influence of the group allocation as an independent binary variable on the primary outcome, namely a potential improvement in grip strength of the (non-) dominant hand(s) from baseline to week 8. For this analysis, each patient was classified as either having improved or not. Any positive change in grip strength was classified as improvement as we expected small to moderate effects. IBM SPSS Statistics for Macintosh (Armonk, NY, USA), Version 24.0 was used for statistical analyses.

## Results

### Demographic data and patient flow

The whole sample was recruited between June 2012 and August 2014. One hundred and seventy-one individuals were informed about the study and were assessed for eligibility. Thereof, three patients declined to participate, six had other reasons for not participating in the study (e.g. long travelling distance to the hospital), and nine patients indicated a pain level < 3 on the day of the baseline assessment and did therefore not fulfil the inclusion criteria. One hundred and fifty-three individuals were randomised; of these, two individuals had a pain level < 3 at the second point in time (day of the intervention) and, therefore, were not included in the study.

**Fig. 2:**
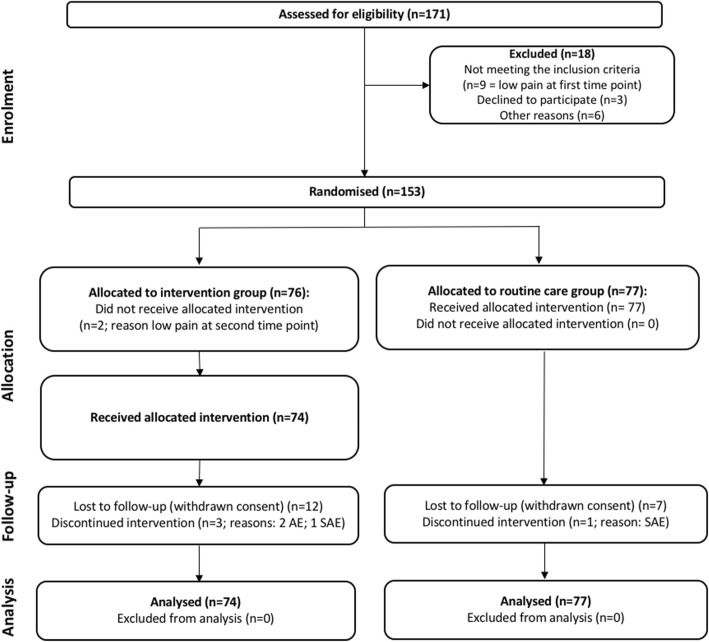
Flow chart displaying the inclusion and randomisation. AE, adverse event; SAE serious adverse event

The 151 patients fulfilling the inclusion/exclusion criteria were randomly assigned to the combined-intervention (*n* = 74) or the routine-care-plus-placebo (*n* = 77) groups (Fig. 2). There were no significant differences in baseline characteristics between the two groups (Table 2).

**Table 2:** Baseline characteristics

Characteristics	All patients	Combined intervention	RC
Demographics			
Patient, *n*	151	74	77
Female, *n* (%)	127 (84)	59 (79.7)	68 (88.3)
Age, mean (SD), years	59.6 (10.6)	60.1 (10.9)	59.1 (10.4)
Disease duration, mean (SD), years	7.6 (9.4)	6.5 (9.2)	9.0 (9.6)
CMC 1 OA (in one or both hands), *n* (%)	75 (50)	36 (48.6)	39 (50.6)
Education (persons obtaining more than compulsory schooling), *n* (%)	74 (49)	37 (50)	37 (48)
Handedness, right handed, *n* (%)	134 (89)	62 (83.8)	72 (93.5)
BMI, mean (SD)	26.3 (4.8)	25.7 (4.4)	26.9 (5.1)
Self-reported satisfaction with appearance of hands on a LS^a^, mean (SD)	1.50 (1.29)	1.47 (1.26)	1.53 (1.32)
Grip strength (Vigorimeter), dominant hand, mean (SD), bar	0.13 (0.19)	0.14 (0.18)	0.13 (0.20)
Grip strength (Vigorimeter), non-dominant hand, mean (SD), bar	0.13 (0.20)	0.14 (0.19)	0.12 (0.21)
Self-reported pain on a LS^a^, mean (SD)	5.16 (2.095)	5.22 (1.96)	5.10 (2.23)
Self-reported satisfaction with treatment on a LS^a^, mean (SD)	7.17 (2.96)	7.26 (2.53)	7.10 (3.30)
Self-reported health status on a LS^a^, mean (SD)	3.99 (2.40)	3.78 (2.35)	4.18 (2.44)
JT Subtest 3, dominant hand, mean (SD)	8.04 (3.82)	7.79 (3.05)	8.28 (4.44)
JT Subtest 3, non-dominant hand, mean (SD)	8.01 (2.75)	7.98 (2.49)	8.05 (2.99)
JT Subtest 7, dominant hand	5.02 (1.49)	5.02 (1.26)	5.02 (1.69)
JT Subtest 7, non-dominant hand, mean (SD)	5.14 (2.23)	5.34 (2.68)	4.94 (1.67)
AUSCAN, mean (SD)	15.71 (4.87)	15.85 (4.08)	15.57 (5.53)

There were no statistically significant differences in baseline characteristics between the combined intervention group and the routine care group

*AUSCAN* Australian/Canadian Hand Osteoarthritis Index, *CMC 1* Carpometacarpal 1 joint, *JT* Jebsen-Taylor Hand Function Test, *OA* osteoarthritis, *RC* routine care

^a^LS = value examined on a Likert scale from 0 to 10

At the baseline assessment, 24 patients in the combined-intervention group and 27 in the routine-care-plus-placebo group indicated they had taken non-steroidal antirheumatics (NSAR). At the follow-up assessment, this number decreased to 13 patients in the combined-intervention group and 18 in the routine-care-plus-placebo group.

The follow-up assessment was completed by 59 participants (77%) in the intervention group and 69 participants (89%) in the routine-care-plus-placebo group: 12 patients in the intervention group and seven in the routine-care-plus-placebo group withdrew consent (Fig. 1). In each group, one serious adverse event occurred: in the intervention group one patient had hand surgery because of pre-existing carpal tunnel syndrome, and in the routine-care-plus-placebo group, one patient had a hand cast applied after an accident. Neither of the adverse events were considered to be related to the study intervention. Furthermore, two adverse events occurred in the intervention group (common cold and tendovaginitis, Additional file 1: Material 2). The tendovaginitis was considered possibly related to the intervention.

### Primary and secondary endpoints

The primary endpoint, grip strength of the dominant hand improved in the combined intervention group and deteriorated in the routine-care-plus-placebo group compared to baseline values; the difference in change from baseline between the two groups at the end of the study was statistically significant (Table 3). A total of 28 patients (38%) improved in the combined-intervention group while in the routine-care group only 15 patients (19%) improved. There were 33 participants in both groups (routine-care group 45%, combined-intervention group 43%) with a grip strength value of 0 at the baseline assessment. At the follow-up assessment, this number increased to 37 (48%) in the routine-care group, but decreased in the combined-intervention group to 17 (23%).

**Table 3:** Effect of intervention: differences within the groups

	Combined intervention difference (FU - BL) (mean (SD))	RC difference (FU - BL) (mean (SD))	*p* value
Grip strength (Vigorimeter), dominant hand, bar	0.03 (0.11)	−0.03 (0.13)	*0.001*
Grip strength (Vigorimeter), non-dominant hand, bar	0.01 (0.10)	−0.03 (0.13)	*0.002*
Self-reported pain on a LS^ab^	−1.35 (2.38)	−0.88 (2.12)	0.339
Self-reported satisfaction with treatment on a LS^ab^	−3.50 (3.37)	− 0.92 (2.95)	*0.002*
Self-reported health status on a LS^ab^	−0.04 (2.00)	−0.44 (2.20)	0.291
JT Subtest 3, dominant hand	−0.55 (1.79)	−0.47 (2.65)	0.193
JT Subtest 3, non-dominant hand	−0.41 (1.96)	0.19 (2.85)	0.010
JT Subtest 7, dominant hand	−0.32 (1.01)	−0.06 (1.30)	0.134
JT Subtest 7, non-dominant hand	−0.39 (1.12)	0.33 (1.27)	*0.000*
AUSCAN^b^	−1.55 (4.95)	−0.63 (4.12)	0.316

Bonferroni adjustment 0.05/10 = 0.005. Mean change - 95% CI - *p*-value

*AUSCAN* Australian/Canadian Hand Osteoarthritis Index, *BL* baseline, *FU* follow up, *JT* Jebsen-Taylor Hand Function Test, *RC* routine care

*p* values set in italics indicate statistical significance

^a^LS = on a Likert scale from 0 to 10

^b^Information on both hands is shown due to how the test/questionnaire is administered

Among the secondary outcomes, there was significant improvement in self-reported satisfaction with treatment and in the JTHFT subtests 7 for the non-dominant hand. There was no significant difference between the groups in change in the JTHFT subtest 3 for both hands and subtest 7 for the dominant hand, in the total AUSCAN score or in change in self-reported pain and health status.

The binominal univariate logistic regression models showed a significant contribution of the treatment/group allocation to the primary outcome (improved versus non-improved, model 1, *p* = 0.012; model 2, *p* = 0.005). The Nagelkerke R square (Table 4) indicated how much of the total variance was explained by each model. The chance to improve grip strength was 2.572 higher in model 1 (dominant hand) and 3.282 higher for model 2 (non-dominant hand) in the intervention group compared to the controls.

**Table 4:** Binominal logistic regression models

#	Primary outcome grip strength Vigorimeter	Nagelkerke R square	Significance	Odds ratio	Confidence interval
Model 1	Dominant hand	0.062	0.012	2.572	1.233–5.365
Model 2	Non-dominant hand	0.085	0.005	3.282	1.439–7.485

To obtain information about the compliance in the combined intervention group, we asked the patients to bring the therapy putty (used to perform the exercises) to the second assessment. After performing the follow-up assessment, assessors scored traces of usage in the therapy putty. In the combined intervention group, four patients (5%) had not used the therapy putty at all according to the judgement of the assessor. In 28 patients (38%), the therapy putty showed substantial traces of usage; the other patients did not bring the therapy putty to the second appointment. However, the combined intervention did not only consist of hand exercises with therapy putty, but also involved functional exercises and exercises without therapy putty; we therefore included all patients in the analysis.

Patients not completing the study had lower grip strength values for both hands and a higher AUSCAN index (meaning poorer function) at baseline (Additional file 1: Material 4). This was seen in both treatment groups.

## Discussion

The combined intervention in our study included several domains that may be important to individual patients. Combined interventions have the advantage of taking individual issues of patients into account. Patients are presented all options and can then make an informed decision about their preferences, main concerns and symptoms. “Exercise-only interventions” for example may not reach patients whose primary concern is not physical activity or loss of function, but pain and aesthetic changes related to hand OA. Combined interventions may be an option to cover all necessary issues and allow patients to set individual priorities. Furthermore, the effect of the intervention may be due to one or two or more components of the combined intervention. Individual combinations of components of interventions could maximize treatment effects without having an approach of “one intervention fits all” and without having to determine precise profiles/subgroups of patients who benefit most from the specific intervention components, e.g. active exercises. The effects of interventions such as exercises, joint protection, self-management strategies or orthoses in hand OA trials are often studied together, as they were in our study protocol [15, 24, 37]. Another approach was taken by Dziedizic et al. who tested the effect of independent components of hand OA interventions with a positive outcome for joint protection [38].

In our study, we developed a time-efficient and personnel-efficient standardised intervention that could be delivered by different non-physician health professionals who were all trained in rheumatology. Our intervention was standardised and was delivered according to a protocol (Table 1) that can be used by health professionals in primary and specialist care settings. Given the number of patients with hand OA and the limited clinical resources, having an intervention that may be delivered by multiple health professionals could be very beneficial. One individual session might have the advantage, compared to a group session, that health professionals can focus explicitly on the needs of the patient and tailor the information according to the patient’s personal needs.

Grip strength was chosen as the primary endpoint because strength is an integral part of hand function, although other aspects may be equally relevant [15, 39, 40]. Osteras et al. concluded that further studies should focus on optimal grip strength exercises [41]. In another RCT, significant improvement in grip strength and activity performance was attained with a home-based hand exercise programme for hand OA [42]. On the other hand, a study involving a multidisciplinary group-based treatment for patients with hand OA showed no effect on grip strength and other outcomes, potentially due to a non-directive approach (patients should select and also develop their own treatment goals and treatment plans) [24].

The difference in grip strength compared to baseline in the intervention group was smaller than expected; however, we consider this result to be clinically significant for three reasons: (1) grip strength deteriorated in the control group over the time period more than it improved in the intervention group, (2) non-pharmacological interventions in rehabilitation in general produce small effects and (3) an intervention with a low, but for the patients acceptable (and satisfying) intensity such as our combined intervention may be used to stabilize rather than to largely improve grip strength.

Some of our secondary outcomes did not show significant effects. The self-reported questionnaire for function probably assesses items that are not relevant to all patients, and some assessed activities are important for selected patients only. While pain may be the main concern of one patient, loss of function may be more important for other patients. Personalized outcome measures, such as the Canadian Occupational Performance Measure [43] in which patients can select certain activities for some domains - e.g. self-care, productivity and leisure - may be an option in future clinical trials, especially if the intervention is tailored to improve occupational performance. There was a reduction in self-reported pain between baseline and follow-up examinations in both groups (Table 3). One possible explanation for this finding may be the greater attention received by patients in both groups.

The results from the two logistic models also confirmed that participants in the combined intervention group had a greater chance of improving grip strength. However, a large number of participants in both groups had a grip strength value of 0 in both the baseline assessment and in follow up. It remains an important issue for further research as to how this group of participants can be effectively treated.

The drop-out rate in the routine-care-group was lower compared to the combined intervention group. This could be related to the fact that the combined intervention was offered to the patients in the routine-care group after the second assessment and that the symptoms in the control group did not improve much with the placebo intervention. Therefore, there may have been greater motivation to return for another assessment.

Existing guidelines and standards of care are frequently not implemented. Our intervention was based on the EUMUSC.net standards of care for OA and can therefore be seen as an example of how the standards can be implemented.

There is currently no disease-modifying drug available for hand OA, and to our knowledge, there is no gold standard design for a programme of non-pharmacological interventions (intervention selection, duration and the intensity/frequency). We focused on interdisciplinary care. To this end, health professionals were trained to cover parts of the interventions that were developed by professionals from other health professions, e.g. physiotherapists and nurses were trained to custom-fabricate a thumb splint. In addition, the intervention was delivered by two health professionals present at the same time to ensure a well-coordinated, effective intervention in different areas of expertise.

While a strength of our study is the detailed and feasible treatment approach for hand OA, our study has limitations as well: one is the choice of instrument to measure grip strength, which had floor effects. Another is that the presence of two health professionals to deliver the intervention might reduce cost-effectiveness. Third, it is evident that due to the type of study, the blinding of patients and the professionals delivering the intervention is lacking. We cannot fully eliminate the possibility that this might have influenced our results e.g. the self-reported outcome measures completed by the patient. However, assessments were made by blinded assessors, the primary outcome was grip strength and data were analysed by the first and last authors, who were not involved in randomisation, nor in the assessment of the patients.

In our study, we applied the combined intervention to patients with finger and/or thumb symptoms. As the aetiology of finger and thumb symptoms may also differ, the response to treatment could also be diverse. This should be investigated in further research.

## Conclusion

The combined, interdisciplinary, individual, one-session intervention significantly improved grip strength and self-reported satisfaction when compared to treatment with routine care plus placebo. This may be an effective and satisfying time-efficient approach in busy clinical settings in both primary and specialised care, which can be delivered by rheumatology-trained non-physician health professionals.

## Additional file

Additional file 1: Material 1 Pictures of the CMC 1 joint orthosis. Material 2 Overview of adverse events and serious adverse events. Material 3 *P* values for baseline characteristics. Material 4 Baseline characteristics. (DOCX 3970 kb)

## Abbreviations

AUSCAN: Australian/Canadian Hand Osteoarthritis Index
CMC 1: Carpometacarpal 1 joint
JTHFT: Jebsen-Taylor Hand Function Test
OA: Osteoarthritis
RC: Routine care
RCT: Randomised controlled trial
