# Supplementary material for: Functional consultation and exercises improve grip strength in osteoarthritis of the hand – a randomised controlled trial
Source: Arthritis Res Ther. 2018 Nov 9;20:253. doi: 10.1186/s13075-018-1747-0 (PMC6235228; doi:10.1186/s13075-018-1747-0)
Supplement: Supplementary file 1 — Material 1 Pictures of the CMC 1 joint orthosis. Material 2 Overview of adverse events and serious adverse events. Material 3 P values for baseline characteristics. Material 4 Baseline characteristics. (DOCX 3970 kb) [file 13075_2018_1747_MOESM1_ESM.docx]

**Additional file 1**

**Material 1** Pictures of the CMC 1 joint orthosis

**Material 2** Overview of adverse events and serious adverse events

| **#** |  | **Event** | **Group allocation** | **Related to intervention** | **Outcome** |
| --- | --- | --- | --- | --- | --- |
| 1 | SAE | Hand Surgery (Carpal Tunnel Syndrome) | Combined Intervention | Not related | Resolved without sequelae |
| 2 | SAE | Cast of the arm | RC | Not related | Resolved without sequelae |
| 1 | AE | Common cold | Combined Intervention | Not related to intervention | Resolved without sequelae |
| 2 | AE | Tendovaginitis | Combined Intervention | Possibly related to intervention | Resolved without sequelae |

AE adverse event, RC Routine Care, SAE serious adverse event;

**Material 3** P-values for baseline characteristics

|  | **All patients** | **Combined Intervention** | **RC** | **p-value** |
| --- | --- | --- | --- | --- |
| Age, mean (SD), years | 59.6 (10.6) | 60.1  (10.9) | 59.1 (10.4) | 0.569 |
| Disease duration, mean (SD), years | 7.6 (9.4) | 6.5 (9.2) | 9.0 (9.6) | 0.077 |
| CMC 1 OA (in one or both hands), n (%) | 75 (50) | 36 (48.6) | 39 (50.6) | 0.861 |
| Education (persons obtaining more than compulsory schooling), n (%) | 74 (49) | 37 (50) | 37 (48) | 0.233 |
| Handedness, right handed, n (%) | 134 (89) | 62 (83.8) | 72 (93.5) | 0.057 |
| BMI, mean (SD) | 26.3 (4.8) | 25.7 (4.4) | 26.9 (5.1) | 0.192 |
| Self-reported satisfaction with appearance of hands on a LS*, mean (SD) | 1.50 (1.29) | 1.47 (1.26) | 1.53 (1.32) | 0.801 |
| Grip strength (Vigorimeter), dominant hand, mean (SD), bar | 0.13 (0.19) | 0.14 (0.18) | 0.13 (0.20) | 0.432 |
| Grip strength (Vigorimeter), non-dominant hand, mean (SD), bar | 0.13 (0.20) | 0.14 (0.19) | 0.12 (0.21) | 0.233 |
| Self-reported pain on a LS*, mean (SD) | 5.16 (2.095) | 5.22 (1.96) | 5.10 (2.23) | 0.574 |
| Self-reported satisfaction with treatment on a LS*, mean (SD) | 7.17 (2.96) | 7.26 (2.53) | 7.10 (3.30) | 0.624 |
| Self-reported health status on a LS*, mean (SD) | 3.99 (2.40) | 3.78 (2.35) | 4.18 (2.44) | 0.303 |
| JT Subtest 3, dominant hand, mean (SD) | 8.04 (3.82) | 7.79 (3.05) | 8.28 (4.44) | 0.505 |
| JT Subtest 3, non-dominant hand, mean (SD) | 8.01 (2.75) | 7.98 (2.49) | 8.05 (2.99) | 0.618 |
| JT Subtest 7, dominant hand | 5.02 (1.49) | 5.02 (1.26) | 5.02 (1.69) | 0.396 |
| JT Subtest 7, non-dominant hand, mean (SD) | 5.14 (2.23) | 5.34 (2.68) | 4.94 (1.67) | 0.118 |
| AUSCAN, mean (SD) | 15.71 (4.87) | 15.85 (4.08) | 15.57 (5.53) | 0.722 |

AUSCAN Australian/Canadian Hand Osteoarthritis Index, CMC 1 Carpometacarpal 1 joint,

JT Jebsen-Taylor-hand-function-test, OA Osteoarthritis, RC Routine Care;

* LS = value examined on a Likert scale from 0-10

There were no statistically significant differences in baseline characteristics between the combined intervention group and the routine care group

**Material 4** Baseline Characteristics

Patients not completing the study showed lower grip strength values for both hands and higher AUSCAN index (meaning poorer function) at baseline. This was seen in both treatment groups.

| **Characteristics** | **All patients** | **Combined Intervention** | **RC** | **Completer Combined Intervention** | **Completer RC** | **Non- completer Combined Intervention** | **Non- completer RC** | **p-value^+^** |
| --- | --- | --- | --- | --- | --- | --- | --- | --- |
| Patient, n | 151 | 74 | 77 | 59 | 69 | 15 | 8 | - |
| Age, mean  (SD), years | 59.6 (10.6) | 60.1 (10.9) | 59.1 (10.4) | 59.4 (10.7) | 58.8 (10.2) | 62.7 (11.7) | 61.6 (12.01) | 0.223 |
| Disease duration, mean (SD), years | 7.6 (9.4) | 6.5 (9.2) | 9.0 (9.6) | 5.5 (6.9) | 9.8 (14.5) | 8.7 (9.4) | 12 (12.6) | 0.668 |
| CMC 1 OA (in one or both hands), n (%) | 75 (50) | 36 (24) | 39  (26) | 29  (49) | 35  (51) | 7  (47) | 4  (50) | 0.706 |
| Education (persons obtaining more than compulsory schooling), n (%) | 74 (49) | 37 (24) | 37  (24) | 30  (51) | 34  (49) | 7  (47) | 3  (37) | 0.651 |
| Handedness, right handed, n (%) | 134 (89) | 62 (41) | 72  (48) | 51  (86) | 64  (93) | 11  (73) | 8  (100) | 0.317 |
| BMI, mean (SD) | 26.3 (4.8) | 25.7 (4.4) | 26.9 (5.1) | 25.7 (4.4) | 26.7 (5.2) | 25.8 (4.8) | 28.6 (3.2) | 0.500 |
| Self-reported satisfaction with appearance of hands on a LS*, mean (SD) | 1.50 (1.29) | 1.47 (1.26) | 1.53 (1.32) | 1.54 (1.27) | 1.58 (1.32) | 1.14 (1.23) | 1.12 (1.36) | 0.140 |
| Grip strength (Vigorimeter), dominant hand, mean (SD), bar | 0.13 (0.19) | 0.14 (0.18) | 0.13 (0.20) | 0.17 (0.19) | 0.14 (0.21) | 0.04 (0.06) | 0.03 (0.05) | **0.018** |
| **Characteristics** | **All patients** | **Combined Intervention** | **RC** | **Completer Combined Intervention** | **Completer RC** | **Non- completer Combined Intervention** | **Non- completer RC** | **p-value^+^** |
| Grip strength (Vigorimeter), non-dominant hand, mean (SD), bar | 0.13 (0.20) | 0.14 (0.19) | 0.12 (0.21) | 0.17 (0.20) | 0.13 (0.21) | 0.02 (0.06) | 0.06 (0.09) | **0.014** |
| Self-reported pain on a LS*, mean (SD) | 5.16 (2.095) | 5.22 (1.96) | 5.10 (2.23) | 5.22 (1.96) | 5.10 (2.26) | 5.20 (2.04) | 5.12 (2.10) | 0.836 |
| Self-reported satisfaction with treatment on a LS*, mean (SD) | 7.17 (2.96) | 7.26 (2.53) | 7.10 (3.30) | 7.46 (2.10) | 7.24 (3.27) | 6.80 (3.42) | 6.00 (3.74) | 0.401 |
| Self-reported health status on a LS*, mean (SD) | 3.99 (2.40) | 3.78 (2.35) | 4.18 (2.44) | 3.83 (2.33) | 4.14 (2.39) | 3.60 (2.50) | 4.5 (2.98) | 0.805 |
| JT Subtest 3, dominant hand, mean (SD) | 8.04 (3.82) | 7.79 (3.05) | 8.28 (4.44) | 7.54 (2.41) | 8.32 (4.78) | 8.31 (4.63) | 7.89 (1.80) | 0.507 |
| JT Subtest 3, non-dominant hand, mean (SD) | 8.01 (2.75) | 7.98 (2.49) | 8.05 (2.99) | 7.80 (1.87) | 7.93 (2.99) | 8.72 (4.13) | 9.12 (2.99) | 0.550 |
| JT Subtest 7, dominant hand | 5.02 (1.49) | 5.02 (1.26) | 5.02 (1.69) | 4.93 (1.13) | 4.98 (1.55) | 5.37 (1.67) | 5.34 (2.84) | 0.665 |
| JT Subtest 7, non-dominant hand, mean (SD) | 5.14 (2.23) | 5.34 (2.68) | 4.94 (1.67) | 5.07 (1.23) | 4.88 (1.52) | 6.44 (5.45) | 5.51 (2.79) | 0.421 |
| AUSCAN, mean (SD) | 15.71 (4.87) | 15.85 (4.08) | 15.57 (5.53) | 15.63 (4.04) | 15.12 (5.46) | 16.81 (4.26) | 19.94 (4.39) | **0.026** |

AUSCAN Australian/Canadian Hand Osteoarthritis Index, CMC 1 Carpometacarpal 1 joint, JT Jebsen-Taylor-hand-function-test, OA Osteoarthritis, RC Routine Care;

LS = value examined on a Likert scale from 0-10

+ p-value refers to the difference between the group of completer vs non-completer
